# Impact of frailty and prefrailty on the mid-term outcomes and rehabilitation course after cardiac surgery

**DOI:** 10.1007/s00595-024-02807-z

**Published:** 2024-03-04

**Authors:** Tasuku Honda, Hirohisa Murakami, Hiroshi Tanaka, Yoshikatsu Nomura, Toshihito Sakamoto, Naomi Yagi

**Affiliations:** 1 Department of Cardiovascular Surgery, Hyogo Prefectural Harima-Himeji General Medical Center, 3-264, Kamiya-Cho, Himeji, Hyogo 670-8560 Japan; 2 Advanced Medical Engineering Research Institute, University of Hyogo, 3-264, Kamiya-Cho, Himeji, Hyogo 670-0836 Japan

**Keywords:** Frailty, Cardiac surgery, Mortality, Morbidity, Rehabilitation

## Abstract

**Purpose:**

This study examined the impact of frailty and prefrailty on mid-term outcomes and rehabilitation courses after cardiac surgery.

**Methods:**

A total of 261 patients (median age: 73 years; 30% female) who underwent elective cardiac surgery were enrolled in this study. The Japanese version of the Cardiovascular Health Study Frailty Index classified 86, 131, and 44 patients into frailty, prefrailty, and robust groups, respectively. We examined the recovery of walking ability, outcomes at discharge, mid-term all-cause mortality, and rehospitalization related to major adverse cardiovascular and cerebrovascular events (MACCE) across the three cohorts.

**Results:**

The 3-year survival rates in the frailty, prefrailty, and robust groups were 87%, 97%, and 100%, respectively (*p* = 0.003). The free event rates of all-cause mortality and re-hospitalization related to MACCE were 59%, 79%, and 95%, respectively (*p* < 0.001), with a graded elevation in adjusted morbidity among patients in the prefrailty (hazard ratio [HR], 4.57; 95% confidence interval [CI], 1.08–19.4) and frailty (HR, 9.29; 95% CI 2.21–39.1) groups. Patients with frailty also experienced a delayed recovery of walking ability and a reduced number of patients with frailty were discharged home.

**Conclusion:**

Frailty and prefrailty adversely affect the mid-term prognosis and rehabilitation course after cardiac surgery.

## Introduction

Frailty is an increase in clinically perceived vulnerability due to a decline in several physiological functions associated with aging which reduces the ability to cope with daily living and acute stress [1]. Frailty is common in older people and carries an increased risk of poor health outcomes including falls, incident disability, hospitalization, and mortality [1]. According to the World Health Organization, the number of people older than 80 years of age is expected to increase three-fold between 2020 and 2050 [2], and the prevalence of frailty is also likely to increase [3].

Frailty commonly coexists with cardiovascular disease, which can lead to poor clinical outcomes. Therefore, frailty has been identified as a risk factor for mortality due to cardiovascular disease [4]. Frailty, which is found in 25–50% of patients undergoing cardiac surgery, is also associated with postoperative functional decline and increased mortality and morbidity from major adverse cardiac and cerebrovascular events (MACCE) [5]. Additionally, frailty has been recognized as a predictor of critical postoperative outcomes after cardiac surgery, prompting the indispensability of preoperative assessment using appropriate tools [6–8].

Nevertheless, frailty is not yet an established prognostic factor following cardiac surgery and preoperative assessment is not commonly performed. Moreover, the relevant literature has limited information on prefrailty which is an intermediary phenotype of frailty, and its precise impact on rehabilitation, mortality, and morbidity after cardiac surgery.

Considering these factors, we hypothesized that both frailty and prefrailty would detrimentally influence the postoperative course, mortality, and morbidity of elderly patients undergoing cardiac surgery. Consequently, this study aimed to examine the effects of frailty and prefrailty on the postoperative course and mid-term prognosis of elderly patients after cardiac surgery.

## Methods

### Study design and subjects

This single-center, prospective, observational study included patients who underwent cardiac surgery at the Department of Cardiovascular Surgery, Hyogo Brain and Heart Center, Japan. This study was conducted following the guidelines established by the Strengthening the Reporting of Observational Studies in Epidemiology and in compliance with the recommended standards for reporting observational studies in epidemiology.

Between August 2018 and November 2021, 500 patients of ≥ 65 years of age who were scheduled for elective coronary artery bypass grafting, valve replacement/repair, thoracic aortic surgery, or other open-heart surgeries were enrolled in this study. All patients underwent a median sternotomy. The exclusion criteria were emergency or urgent surgery, descending or thoracoabdominal aortic surgery performed with left intercostal thoracotomy, difficulty walking related to functional disorders, and incomplete data. Postoperative rehabilitation, functional recovery programs, and exercise intensity standards were based on the Japanese Cardiovascular Society guidelines [9]. The participants were followed postoperatively using medical records and telephone interviews. The date of the last follow-up for survival or death was January 2023. Informed consent was obtained from all participants and an opt-out strategy ensured the opportunity to refuse to participate in the study. This study was approved by the Research Ethics Committee of the Hyogo Brain and Heart Center under protocol number R3-27 and was conducted in accordance with the Declaration of Helsinki.

### Frailty assessment

We selected the Japanese version of the Cardiovascular Health Study (J-CHS) frailty index [10] that defines “frailty” as having three or more of the following: (1) weight loss of ≥ 2 kg in the past 6 months; (2) exhaustion (“In the past 2 weeks, have you felt tired without reason?”); (3) Low activity (Engages in moderate levels of physical exercise or sports aimed at health and engages in low levels of physical exercise aimed at health); (4) slowness (gait speed < 1.0 m/s); and (5) weakness (grip strength < 28 kg in men or < 18 kg in women). To calculate gait speed, we asked the patients to walk at a comfortable pace on a designated 5-m walkway and measured the time taken to complete the course. Patients who met one or two criteria were classified as having “prefrailty,” and those who did not meet any of the criteria were classified as “robust.”

### Data collection

We collected the following data: age, sex, body mass index (BMI), skeletal muscle mass index (SMI) as measured by a bioelectrical impedance analysis, geriatric nutritional risk index (GNRI), left ventricular ejection fraction (LVEF), percent vital capacity (%VC), forced expiratory volume 1.0 s percent (FEV1%), predicted mortality calculated from the European System for Cardiac Operative Risk Evaluation (EuroSCORE) II [11], the JapanSCORE, which was devised as an original Japanese risk model for cardiovascular surgery [12]; and the presence of sarcopenia, dyslipidemia, diabetes mellitus, hypertension, chronic kidney disease, and cerebrovascular disease (CVD). Sarcopenia was diagnosed based on the criteria reported by the Asian Working Group [13]. Moreover, we documented the type and duration of surgery, blood loss volume, blood transfusion volume, postoperative intubation time, length of ICU stay, day of initiation of postoperative rehabilitation, progress of gait training, new-onset atrial fibrillation or CVD during hospitalization, length of hospital stay after cardiac surgery, and whether the patient was discharged or transferred to another facility. In this study, delayed rehabilitation was defined as rehabilitation that could not be performed the day after surgery due to reoperation, complications, or the absence of a therapist. These data were gathered retrospectively from medical records.

### Clinical outcomes

The primary outcome was all-cause mortality from the date of the index surgical procedure to 3 years after the surgical procedure in the frailty, prefrailty, and robust groups. The secondary outcomes were morbidity at 3 years postoperatively, which consisted of all-cause mortality and MACCE-related rehospitalization from acute myocardial infarction, heart failure, and stroke. The third outcome was the postoperative recovery of walking ability and whether the patient could be discharged.

### Statistical analysis

Continuous variables were presented as medians with interquartile ranges (first–third quartiles). Categorical variables were expressed as numbers (n) and proportions (%). We used the Kruskal–Wallis test to compare the averages of continuous variables (such as age) and the chi-square test to compare the proportion of categorical variables (such as sex) among groups.

Analyses of preoperative characteristics and postoperative outcomes among the groups were first performed using analysis of variance (ANOVA). If there was a significant difference, multiple comparisons with Bonferroni correction were conducted. Kaplan–Meier curves were plotted for all-cause mortality and morbidity (all-cause mortality and rehospitalization-related MACCE) in the three groups. Three-sample log-rank tests were applied to test for differences in cumulative event rates. Using a Cox proportional hazards model, we calculated the hazard ratio of the Kaplan–Meier curve for each group. If there were significant differences in patient characteristics propensity score matching was performed to adjust prognostic factors between the groups.

Missing values were managed using the common category for categorical covariates, and conditional medians for continuous covariates.

All statistical analyses were performed using the IBM SPSS Statistics software (version 29.0; IBM Corp., Armonk, NY, USA). All *p*-values were two-sided and p values of < 0.05 were considered to indicate statistical significance.

## Results

**Fig. 1:**
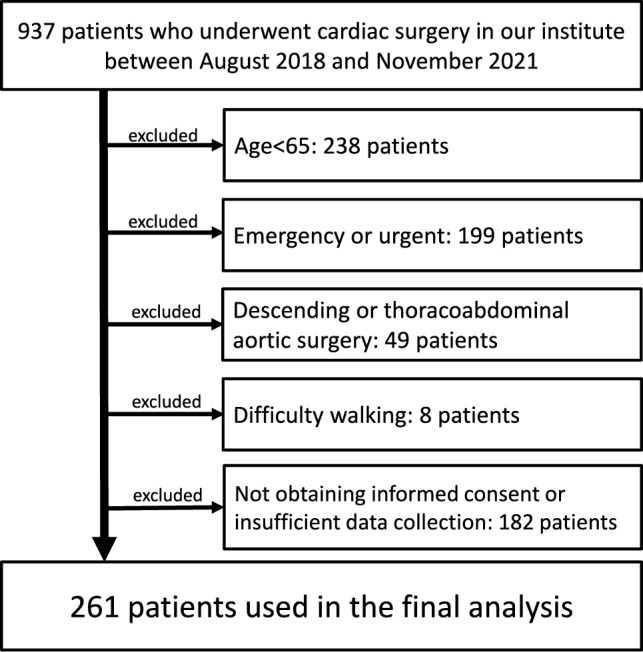
Flow diagram of study enrollment

Overall, 261 patients (median age, 73 years; 30% women) who underwent both elective cardiac surgery and preoperative frailty assessment were enrolled in this study (Fig. 1). The J-CHS Frailty Index classified 86 (33%), 131 (50%), and 44 (17%) patients into the frailty, prefrailty, and robust groups, respectively. Table 1 shows the number of patients in the three groups who fulfilled each of the five J-CHS items, preoperative gait speed, and grip strength by sex. The results revealed significant differences among the three groups for all items. The median follow-up duration was 1181 days (first–third quartile: 883–1432 days), and all surviving patients were followed for at least 3 years.

**Table 1:** Frailty assessment items (J-CHS frailty index)

	Overall (*n* = 261)	Frailty group (*n* = 86)	Prefrailty group (*n* = 131)	Robust group (*n* = 44)	*p* value
Weight loss	75 (29)	47 (55)	28 (21)	0 (0)	p < 0.001
Exhaustion	67 (26)	46 (54)	21 (16)	0 (0)	p < 0.001
Low activity	155 (59)	77 (90)	78 (60)	0 (0)	p < 0.001
Slowness*	123 (47)	67 (78)	56 (43)	0 (0)	p < 0.001
Gait speed (m/s)	1.0 [0.9–1.2]	0.9 [0.8–1.0]	1.0 [0.9–1.2]	1.2 [1.1–1.3]	p < 0.001
Weakness**	69 (26)	53 (62)	16 (12)	0 (0)	p < 0.001
Male grip strength (kg)	32 [27–36]	27 [22–32]	33 [30–37]	33 [29–36]	p < 0.001
Female grip strength (kg)	18 [15–21]	16 [14–18]	19 [17–22]	21 [19–23]	p < 0.001

Data are presented as the median [interquartile range] or n (%)

J-CHS: Japanese version of the Cardiovascular Health Study

^*^ Gait speed < 1.0 m/s ** Grip strength < 28 kg (male), < 18 kg (female)

### Baseline characteristics and operative data

Table 2 shows the baseline characteristics of each group. There were significant differences among the three groups in age, sex, BMI, SMI, sarcopenia, GNRI, malnutrition (GNRI < 92), hemoglobin, serum albumin, low serum albumin (≤ 3.5 *g*/dl), and predictive operative mortality calculated by JapanSCORE. Meanwhile, the preoperative LVEF, %VC, FEV1%, EuroSCORE II, and mortality and morbidity calculated using JapanSCORE did not differ to a statistically significant extent among the groups. Regarding operative data, with the exception of the surgical procedure category, there were no significant differences in operation time, blood loss, or transfusion volume (Table 3).

**Table 2:** Baseline characteristics

	Overall (*n* = 261)	Frailty group (*n* = 86)	Prefrailty group (*n* = 131)	Robust group (*n* = 44)	*p* value
Age (years)	73 [70–77]	76 [72–80]	72 [68–76]	73 [68–78]	0.012
Female	79 (30)	41 (48)	31 (24)	7 (16)	p < 0.001
Body mass index (kg/m^2^)	22.6 [20.3–24.9]	21.4 [18.9–23.9]	23.0 [20.7–25.4]	23.0 [21.0–25.1]	0.016
SMI (kg/m^2^)	7.0 [6.0–7.6]	6.4 [5.5–7.2]	7.2 [6.5–7.8]	7.2 [6.6–7.7]	p < 0.001
Sarcopenia	53 (20)	36 (42)	17 (13)	0 (0)	p < 0.001
GNRI	102 [96–109]	98 [91–105]	104 [97–110]	104 [100–109]	p < 0.001
Malnutrition (GNRI < 92)	40 (15)	26 (30)	12 (9)	2 (5)	p < 0.001
Dyslipidemia	115 (44)	39 (45)	60 (46)	16 (36)	0.540
Diabetes mellitus	67 (26)	26 (30)	31 (24)	10 (23)	0.524
Hypertension	205 (79)	69 (80)	105 (80)	31 (71)	0.381
Chronic kidney disease	79 (30)	26 (30)	38 (29)	15 (34)	0.787
Hemoglobin (g/dl)	13.4 [12.3–14.5]	12.7 [11.5–13.8]	13.6 [12.5–14.7]	13.4 [12.4–14.4]	0.002
Serum albumin (g/dl)	4.0 [3.7–4.2]	3.8 [3.5–4.1]	4.0 [3.8–4.3]	4.1 [3.9–4.3]	p < 0.001
Low serum albumin (≤ 3.5 g/dl)	45 (17)	25 (29)	16 (12)	4 (9)	0.002
LVEF (%)	61 [52–65]	60 [55–66]	62 [54–70]	60 [55–66]	0.623
%VC	93 [84–103]	91 [78–104]	95 [86–104]	91 [83–99]	0.182
%FEV_1_	77 [70–82]	77 [71–83]	76 [71–81]	78 [71–85]	0.657
Prior history of CVD	93 (36)	30 (35)	48 (37)	15 (34)	0.988
EuroSCORE II(%)	2.7 [1.7–4.1]	3.1 [1.7–4.4]	2.4 [1.2–3.7]	2.5 [1.4–3.7]	0.124
JapanSCORE; mortality (%)	3.3 [1.8–4.8]	3.8 [2.4–5.5]	3.2 [1.6–4.0]	3.2 [1.2–4.0]	0.015
JapanSCORE; mortality and morbidity (%)	15.0 [10.8–22.7]	17.7 [12.9–24.3]	14.3 [10.4–21.3]	14.3 [10.0–23.3]	0.069

Data are presented as the median [interquartile range] or n (%)

*SMI* skeletal muscle mass index, *GNRI* geriatric nutritional risk index, *LVEF* left ventricle ejection fraction, *%VC* percent vital capacity, *% FEV*_*1*_ forced expiratory volume 1.0 s percent, *CVD* cerebrovascular disease, *EuroSCORE* the European System for Cardiac Operative Risk Evaluation

**Table 3:** Operative data

	Overall (*n* = 261)	Frailty group (*n* = 86)	Prefrailty group (*n* = 131)	Robust group (*n* = 44)	*p* value
Operation category	CABG: 43 Valve: 106 Aorta: 106 Others: 7	CABG: 11 Valve: 47 Aorta: 24 Others: 4	CABG: 22 Valve: 45 Aorta: 63 Others: 1	CABG: 10 Valve: 14 Aorta: 18 Others: 2	0.011
Off-pump CABG	14 (5)	5 (6)	6 (5)	3 (7)	0.812
Concomitant surgery	61 (23)	24 (28)	31 (24)	6 (14)	0.193
Operation time (min)	279 [245–326]	263 [216–310]	290 [246–335]	271 [247–296]	0.070
CPB time* (min)	147 [125–169]	143 [118–169]	152 [127–176]	142 [125–158]	0.282
Clamp time* (min)	93 [74–118]	98 [79–117]	92 [69–115]	88 [57–119]	0.305
Blood loss volume (ml)	300 [190–480]	320 [179–462]	290 [130–450]	300 [180–420]	0.694
RBC transfusion (unit)	4 [2–6]	6 [3–9]	4 [2–6]	4 [1–7]	0.115
FFP transfusion (unit)	6 [2–10]	6 [1–11]	6 [0–12]	6 [2–10]	0.639
PC transfusion (unit)	10 [5–15]	10 [5–15]	10 [5–15]	10 [5–15]	0.550

Data are presented as the median [interquartile range] or n (%)

*CABG* coronary artery bypass grafting, *CPB* cardio-pulmonary bypass, *RBC* red blood cell, *FFP*　fresh frozen plasma, *PC* platelet concentrate

^*^Excluding 14 cases in which extracorporeal circulation was not used

### Primary outcome: mortality

There were 13 deaths during the observation period and the etiologies included CVD in 3 patients, pneumonia in 2 patients, and heart failure, aortic dissection, cancer, and sepsis in 1 patient each. The cause of death remained undetermined in 4 patients.

**Fig. 2:**
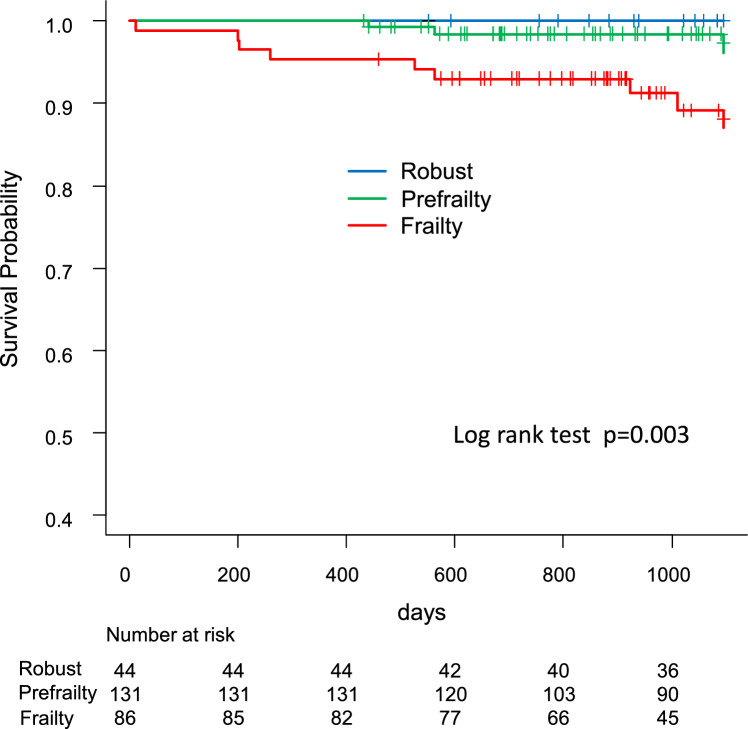
Kaplan–Meier curves for all-cause death according to the frailty assessment

Figure 2 shows the Kaplan–Meier curves for all-cause death in all groups. The 3-year survival rates in the frailty, prefrailty, and robust groups were 87%, 97%, and 100%, respectively. The ANOVA with a log-rank analysis revealed significant differences (*p* = 0.003). Although there was a significant difference between the frailty and prefrailty groups (hazard ratio [HR], 5.05; 95% confidence interval CI 1.36–18.5, *p* = 0.015), comparisons with the other two groups were impractical due to the absence of deaths in the robust group.

### Secondary outcome: morbidity

In this study, 65 MACCE-related rehospitalizations were observed, including 20 patients with worsening heart failure, 13 patients with new arrhythmias, and 11 patients with CVD. Moreover, additional surgery and percutaneous coronary intervention were performed in 6 patients each.

**Fig. 3:**
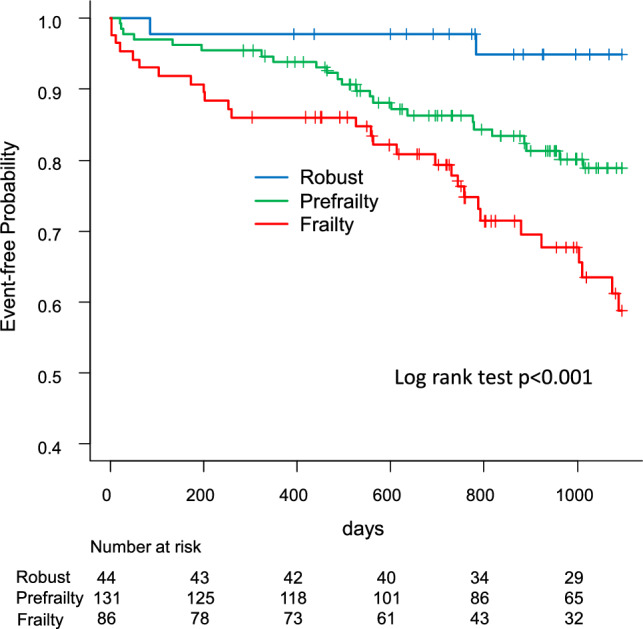
Kaplan–Meier curves for all-cause death and readmission related to major adverse cardiovascular and cerebrovascular events according to the frailty assessment

Figure 3 shows the Kaplan–Meier curves for all-cause death and readmission related to MACCE in all groups. The 3-year event-free rates were 59%, 79%, and 95% in the frailty, prefrailty, and robust groups, respectively (log-rank test, *p* < 0.001). In comparison to the robust group, there was a graded increase in adjusted morbidity among patients in the prefrailty (HR, 4.57; 95% CI 1.08–19.4, *p* = 0.039) and frailty (HR, 9.29; 95% CI 2.21–39.1, *p* < 0.001) groups.

### Propensity score analysis

We performed a propensity score analysis for matching because there were significant age and sex differences related to life expectancy among the three groups. Patients were matched into frailty and non-frailty (prefrailty and robust) groups. As a result, 76 patients were selected for each group. After adjusting for age and sex, frailty was associated with higher mid-term mortality (adjusted HR, 9.26; 95% CI 1.15–74.34; *p* = 0.036) and morbidity (adjusted HR, 6.81; 95% CI 2.60–17.87; *p* < 0.001) rates (Table 4 and 5). Malnutrition and low serum albumin were also identified as significant risk factors for mid-term morbidity in the adjusted model (Table 4).

**Table 4:** Cox proportional hazards analyses of preoperative variables and mid-term all-cause mortality

Variables	Unadjusted (*n* = 261)			Adjusted for propensity* (*n* = 152)		
	HR	95%CI	*p* value	HR	95%CI	*p* value
Frailty	6.856	1.852–25.381	0.004	9.259	1.153–74.336	0.036
Gait speed	0.114	0.013–1.002	0.050	0.314	0.020–4.899	0.408
Grip strength	0.947	0.883–1.015	0.122	0.977	0.901–1.059	0.565
SMI	0.772	0.471–1.265	0.305	0.934	0.528–1.654	0.816
Sarcopenia	2.767	0.878–8.721	0.082	2.249	0.604–8.376	0.227
Malnutrition (GNRI < 92)	5.778	1.862–17.926	0.002	5.532	1.485–20.616	0.011
Hemoglobin	0.720	0.521–0.994	0.046	0.831	0.577–1.197	0.319
Low serum albumin (≤ 3.5 *g*/dl)	5.144	1.656–15.974	0.005	5.035	1.351–18.768	0.016

^*^After adjusting for age and sex, patients were matched into frailty and non-frailty (prefrailty and robust) groups. Seventy-six patients were selected for each group

*CI* confidence interval, *SMI* skeletal muscle mass index, *GNRI* geriatric nutritional risk index

**Table 5:** Cox proportional hazards analyses of preoperative variables and mid-term all-cause mortality or readmission due to MACCE

Variables	Unadjusted (*n* = 261)			Adjusted for propensity* (*n* = 152)		
	HR	95%CI	*p* value	HR	95%CI	*p* value
Frailty	2.589	1.516–4.420	p < 0.001	6.813	2.597–17.871	p < 0.001
Gait speed	0.797	0.256–2.477	0.695	0.668	0.148–3.009	0.600
Grip strength	0.967	0.937–0.998	0.035	0.962	0.920–1.007	0.094
SMI	0.955	0.756–1.207	0.702	0.989	0.726–1.348	0.946
Sarcopenia	1.486	0.819–2.697	0.192	1.384	0.648–2.958	0.401
Malnutrition (GNRI < 92)	1.575	0.812–3.054	0.179	1.781	0.792–4.003	0.162
Hemoglobin	0.921	0.787–1.079	0.309	0.890	0.726–1.090	0.259
Low serum albumin (≤ 3.5 *g*/dl)	1.801	0.980–3.313	0.058	1.803	0.825–3.938	0.139

^*^After adjusting for age and sex, patients were matched into frailty and non-frailty (prefrailty and robust) groups. Seventy-six patients were selected for each group

*MACCE* Major Adverse Cardiovascular and Cerebrovascular Events, *CI* confidence interval, *SMI* skeletal muscle mass index, *GNRI* geriatric nutritional risk index

### Third outcome: postoperative course

Table 6 shows the postoperative course in each group. Patients in the frailty group had longer intubation times (15 vs. 7 h), slower achievement of 100-m walk distance (4 vs. 3 days), higher rates of 300-m continuous walk impossible (26% vs. 11%), and a significant decrease in home discharge rates (85% vs. 96%). The reasons for transfer to another institute were the need for continued treatment of non-cardiac complications or rehabilitation.

**Table 6:** Postoperative data

	Overall (*n* = 261)	Frailty group (*n* = 86)	Prefrailty group (*n* = 131)	Robust group (*n* = 44)	*p* value
Intubation time (hour)	14 [5–17]	15 [9–21]	14 [8–20]	7 [0–13]	0.018
ICU stay (day)	3 [2–3]	2 [2–4]	3 [2–3]	3 [2–3]	0.630
Start of rehabilitation (POD)	1 [1–1]	1 [1– 2]	1 [1–1]	1 [1–1]	0.703
Late rehabilitation	55 (21)	18 (21)	28 (21)	9 (21)	0.991
Start of walking (POD)	2 [2–4]	3 [2–5]	2 [2–4]	2 [2–3]	0.068
Day of 100-m walk achievement (POD)	3 [2–4]	4 [3–6]	3 [2–4]	3 [2–5]	p < 0.001
300-m continuous walk impossible	40 (15)	22 (26)	13 (10)	5 (11)	p < 0.001
Postoperative AF	84 (32)	30 (35)	40 (31)	14 (32)	0.807
Postoperative CVD	20 (8)	9 (11)	5 (6)	4 (9)	0.326
Admission days after surgery	21 [16–26]	22 [16–28]	21 [17–26]	19 [15–24]	0.563
Discharge to home	239 (92)	73 (85)	124 (95)	42 (96)	0.035
Transfer to another institute	21 (8)	12 (14)	7 (5)	2 (5)	0.048

Data are presented as the median [interquartile range] or *n* (%)

*ICU* intensive care unit, *POD* postoperative day, *AF* atrial fibrillation, *CVD* cerebrovascular disease

## Discussion

To the best of our knowledge, there are no reports that have concurrently examined the rehabilitation course and mid-term prognosis following cardiac surgery in frailty, prefrailty, and robust groups. Based on the results of this study, we identified the following findings: first, frailty and prefrailty were associated with an unfavorable mid-term prognosis after cardiac surgery, irrespective of prognostic predictors such as the EuroSCORE II. Second, even after adjusting for age and sex, the impact of frailty on the mid-term prognosis following cardiac surgery remained consistent. Finally, frailty impeded walking recovery after cardiac surgery, resulting in fewer patients being discharged.

According to a systematic review and meta-analysis that included 66,446 patients, frailty and prefrailty were associated with greater adjusted perioperative complications and adjusted mid-term mortality in patients undergoing cardiac surgery, with correlations of twofold and 1.5-fold, respectively [14]. Another systematic review reported that frailty had a strong positive relationship with the risk of MACCE after cardiac surgery (odds ratio [OR], 4.89; 95% CI 1.64–14.60) [5]. Thus, the findings of this study agree with those of previous reports on the impact of frailty and prefrailty on mid-term outcomes after cardiac surgery. Meanwhile, a previous report revealed higher 1-year mortality rates in the frailty (36%), prefrailty (17%), and robust (8%) groups [15] than our 3-year mortality rates (13%, 3%, and 0%, respectively). Moreover, previous studies have indicated higher postoperative risk predictors in patients with frailty [16]. However, in this study, no significant differences were observed among the three groups in terms of EuroSCORE II and mortality and morbidity calculated by JapanSCORE. This may be attributed to the increased prevalence of minimally invasive procedures, such as transcatheter aortic valve implantation (TAVI), over the past decade which is now widely performed in high-risk patients within groups with frailty. Mortality rates calculated by JapanSCORE were significantly different among the three cohorts, in contrast to the results of the EuroSCORE II. Regarding the reason for this difference, we speculate that JapanSCORE includes BMI as input information, which is not included in EuroSCORE II, and that it indirectly expresses the degree of frailty or nutrition.

Generally, frailty is more prevalent among elderly individuals and women [3, 17], raising concerns about the potential strong influence of frailty on the life prognosis. Consequently, we analyzed mid-term mortality and morbidity after cardiac surgery using propensity score matching based on age and sex. We revealed that the negative impact of frailty on the mid-term prognosis after cardiac surgery remained consistent. Another study reported that the effect of frailty on mortality varied with age and not sex, with mortality decreasing linearly with increasing patient age [18]. Overall, these findings underscore the critical importance of preoperative frailty assessment in patients undergoing cardiac surgery, even those aged < 65 years.

The postoperative course of patients undergoing cardiac surgery is influenced by frailty. Studies suggest that frailty is independently associated with prolonged intubation times, decreased functional status [19], increased discharge to non-home locations [19, 20], and hospital mortality [16]. In this study, we explored the rehabilitation course after cardiac surgery in patients with frailty and prefrailty, with a focus on the recovery of their ability to walk. Consequently, although the frailty group had a longer intubation time, rehabilitation was initiated on the day after surgery, which was similar to that in the other groups. However, the subsequent recovery of walking ability was delayed in the frailty group. We believe that this decline in postoperative walking ability led to transfer to other facilities for continued rehabilitation.

Frailty is one of the most critical variables with a proven impact on the increased risk of morbidity and mortality in cardiac surgery [21]. The incorporation of frailty measures into existing perioperative risk models markedly enhances the predictive performance for mortality [22]. Thus, the importance of assessing frailty before cardiac surgery for risk stratification, prediction of postoperative outcomes, and formulation of appropriate strategies has long been emphasized. However, frailty assessment has not been integrated into routine clinical practice or major risk assessment models such as the EuroSCORE II or Society of Thoracic Surgeons Risk Score [20]. One reason for this is the heterogeneity and complexity of the assessment methods. Since Fried et al. proposed the concept of frailty [23], various tools have been used for its assessment, highlighting the need for standardization and guidelines [24]. The previous meta-analysis included 19 observational studies, of which only one used the CHS criteria proposed by Fried for frailty assessment. The authors also described that the various frailty assessments may have led to prefrailty patients being assigned to either frailty or robust groups across different studies. Real clinical settings continue to rely on non-standardized methods such as the eyeball test. However, this rapid and subjective assessment method lacks the requisite reliability to gauge frailty accurately. Moreover, a consensus on a specific multidimensional tool for assessing frailty in cardiac surgery with a high-risk predictive value has yet to be established [21].

Gait speed is known as the “sixth vital sign” [25], and is a valid, reliable, and sensitive measurement for assessing and monitoring functional status and overall health in a wide range of populations [26]. Afilalo et al. revealed that the 1-year survival rates were 90% (< 0.83 m/s), 95% (0.83–1.00 m/s), and 97% (> 1.00 m/s) in the slow, middle, and fast gait speed tertiles, respectively, and that the risk of hospitalization in these groups was 45%, 33%, and 27% (both *p* < 0.0001) [27]. After adjustment, gait speed remained a significant predictor of mortality (HR, 2.16 per 0.1-m/s decrease in gait speed; 95% CI 1.59–2.93) and re-hospitalization (HR, 1.71 per 0.1-m/s decrease in gait speed; 95% CI 1.45–2.0) [27]. Additionally, gait speed significantly correlates with the 6-min walking distance and is an indicator of difficulty in performing activities of daily living in patients with cardiovascular disease [28]. Therefore, gait speed is a simple screening tool for frailty, with the potential to act as a strong predictor of the postoperative course and mid-term outcomes in patients following cardiac surgery.

To our knowledge, no specific study has explored the effects of frailty and prefrailty on the rehabilitation course after cardiac surgery. As mentioned previously, high-risk cases within the frailty group in this study were more likely to undergo minimally invasive procedures such as TAVI. Additionally, the date on which rehabilitation was initiated did not differ to a statistically significant extent in our groups. Nonetheless, the recovery of postoperative walking ability was delayed in patients in the frailty group. Given the reversible nature of frailty, preoperative rehabilitation is a promising strategy for improving the postoperative course progression. Preoperative home-based exercise programs may present a solution for the decline in physical function after cardiac surgery in patients with frailty [29]. Furthermore, a previous study demonstrated significantly slower postoperative ambulation initiation and lower home discharge rates in malnourished patients [30]. In this study, the frailty group also exhibited a significantly lower SMI and GNRI, as well as a higher rate of sarcopenia. The efficacy of a home-based comprehensive cardiac rehabilitation program that incorporates exercise training and nutritional counseling has been documented [31]. This fuels expectations that preoperative interventions for patients scheduled for cardiac surgery will substantially enhance postoperative physical function and mid-term outcomes.

The present study was associated with several limitations. First, the analysis was conducted at a single institution, which resulted in a limited sample size. Additionally, we excluded patients undergoing urgent cardiac surgery because of the inability to conduct preoperative assessments, such as gait speed measurements in patients in an unstable state. Notably, previous studies have included urgent surgical cases within their cohorts, which may have potentially influenced the postoperative course and prognosis of the patients. Second, there was a notable bias in the distribution of surgical procedures among the three groups. Valve surgery was more prevalent in the frailty group, which may have included a higher proportion of patients with a history of heart failure. However, we believe that the influence of the surgical procedure is relatively minor, given that no significant differences were observed in EuroSCORE II, including the preoperative NYHA classification and other surgical factors. Third, this study did not consider the effects of cognitive impairment or postoperative delirium. The cognitive function is a key element that warrants frailty assessment [32]. The greatest risk of delirium after cardiac surgery reportedly occurs when frailty and mild cognitive impairment coexisted [33]. Additionally, coexisting frailty and postoperative delirium resulted in a 30-fold increased risk of 1-year mortality [34]. These findings suggest that cognitive impairment and postoperative delirium may influence the outcome of patients with frailty, and we believe that an analysis that includes these factors is necessary. Finally, we did not reassess frailty after surgery in this study. A previous study reported that up to 50% of frail patients showed an improved frailty status after surgery [35]. Changes in the frailty status after surgery will be the subject of future studies.

## Conclusion

In conclusion, both frailty and prefrailty have adverse effects on the postoperative course and mid-term prognosis of patients after cardiac surgery, independent of age and sex. These effects persisted even in the current era of minimally invasive surgery, as exemplified by TAVI. Notably, a graded escalation in adjusted mid-term morbidity was evident among patients in the prefrailty (HR, 4.57; 95% CI 1.08–19.4) and frailty (HR, 9.29; 95% CI 2.21–39.1) groups in comparison to the robust group. Additionally, frailty is associated with postoperative decline in walking ability and an elevated rate of non-home discharge.

Preoperative frailty assessment is essential for predicting outcomes after cardiac surgery. Preoperative intervention with comprehensive cardiac rehabilitation, including exercise training and nutritional counseling, is recommended to address the challenges arising from frailty. Further studies are needed to investigate the impact of preoperative home-based rehabilitation in high-risk patients with frailty.
